# Emotional Intelligence in Children with Severe Sleep-Related Breathing Disorders

**DOI:** 10.1155/2019/6530539

**Published:** 2019-09-05

**Authors:** Francesca Felicia Operto, Francesco Precenzano, Ilaria Bitetti, Valentina Lanzara, Maria Lorena Fontana, Grazia Maria Giovanna Pastorino, Marco Carotenuto, Francesco Pisani, Anna Nunzia Polito, Daniela Smirni, Michele Roccella

**Affiliations:** ^1^Child and Adolescent Neuropsychiatry, Medical School, University of Salerno, Italy; ^2^Sleep Lab for Developmental Age, Clinic of Child and Adolescent Neuropsychiatry, Department of Mental Health, Physical and Preventive Medicine, University of Campania “Luigi Vanvitelli”, Napoli, Italy; ^3^IRCCS Centro Neurolesi “Bonino Pulejo”, Messina, Italy; ^4^Child Neuropsychiatry Unit, Medicine & Surgery Department, University of Parma, Italy; ^5^Complex Structure of Neuropsychiatry Childhood-Adolescence of Ospedali Riuniti of Foggia, Foggia, Italy; ^6^Department of Psychology, Educational Science and Human Movement, University of Palermo, Italy

## Abstract

**Background:**

Obstructive sleep apnea syndrome (OSAS) affects up to 4% of a pediatric population, with many comorbidities in the medium-long term. Functional alterations in the prefrontal cortex (PFC) may explain why OSAS impacts aspects such as executive functions, memory, motor control, attention, visual-spatial skills, learning, and mood regulation. Emotional intelligence (EI) is a complex neuropsychological function that could be impaired in many clinical conditions.

**Purpose:**

The aim of the study is to evaluate the difference in emotional intelligence skills among children with OSAS and healthy subjects (nOSAS).

**Methods:**

129 children (72 males; mean age 7.64 ± 1.98 years) affected by OSAS were compared to 264 non-OSAS (nOSAS) children (138 males; mean age 7.98 ± 2.13) similar for gender, age, and socioeconomic status. In order to assess the emotional quotient, the Bar-On Emotional Quotient Inventory: Youth Version (EQ-i:YV) was used.

**Results:**

The comparison for means and standard deviation between OSAS children and nOSAS children for EQ-i:YV scores showed significant differences for Interpersonal, Adaptability, and Stress Management scales and EQ Total score.

**Conclusions:**

Our findings highlighted the role of intermittent hypoxia in the genesis of the effects of sleep-related respiratory disorders, which involves also aspects different from physical impairments.

## 1. Introduction

Sleep-related breathing disorders (SRBD) in children refer to several nocturnal events ranging from habitual snoring to the obstructive sleep apnea syndrome (OSAS) and affecting up to 4% of the pediatric population, particularly between 5 and 7 years.

Such nocturnal respiratory disorders, since childhood and adolescence, may severely impact on cardiovascular functions [1], orofacial thrive [2], and the neuroendocrine and central nervous system [3–6].

Beebe and Gozal [7] described the daytime cognitive and behavioral deficits in children affected by OSAS, actually pinpointed by the evidence of functional alterations in the prefrontal cortex (PFC), in brain tissue integrity, and in grey matter density deficit [8, 9].

On the functional level, OSAS in childhood may impair several neurocognitive functions such as executive functions, memory, motor control, attention, and visual-spatial skills [10, 11]. In addition, OSAS may be associated to emotional and mood dimensions [12–15], autism spectrum disorders [16], primary headaches [17, 18], and epilepsy [19–22].

Mayer and Salovey [23] defined emotional intelligence (EI) as “the ability to perceive accurately, appraise, and express emotion; the ability to access and/or generate feelings when they facilitate thought; the ability to understand emotion and emotional knowledge; and the ability to regulate emotions to promote emotional and intellectual growth”. Therefore, EI may be intended as a complex neuropsychological function that could be impaired in many clinical conditions.

The current study investigated whether the emotional intelligence may be influenced by the intermittent hypoxia and its impact on fronto-prefrontal regions in a sample of children suffering from OSAS. The aim of the study was to evaluate the difference in emotional intelligence skills among children with OSAS and healthy typical developing subjects. We hypothesized that emotional intelligence skills may be impaired in OSAS children, compared to those of typical developing subjects.

## 2. Methods

### 2.1. Study Design

The current study is cross-sectional between groups' designs, involving two groups of children: an OSAS group and a group of non-OSAS.

### 2.2. Population

A sample of 393 children participated in the study. The study sample was subdivided into two groups: (1) the OSAS group, involving 129 children (72 males, 57 females; mean age 7.64 ± 1.98 years) affected by SRBD diagnosed with polysomnographic examination (PSG) in accordance with international criteria, and (2) the nOSAS group, involving 264 children (138 males, 126 females; mean age 7.98 ± 2.13) with typical development.

For both groups, the exclusion criteria were the following: overweight (*z* − BMI > 85 pc) and obesity (*z* − BMI > 95 pc); cognitive disability (IQ < 70); neurological disorders (i.e. headaches and epilepsy); chromosomal syndromes (i.e., Down syndrome, Prader-Willi syndrome, Crouzon syndrome, Pierre-Robin syndrome, and trisomy 18); and psychiatric illnesses (i.e., mood disorders, anxiety disorders, and psychosis). The absence of these conditions was assessed during the first visit by a pediatrician and by a child neuropsychiatrist.

All participants were recruited through the records of children admitted to the Sleep Lab for Developmental Age of the University of Campania “Luigi Vanvitelli.” As part of a usual clinical practice, all patients were administered polysomnographic examination and the Bar-On Emotional Quotient Inventory: Youth Version (EQ-i:YV) self-report questionnaire.

Participants were recruited from the same urban area, they were all of Caucasian origin, and they had middle socioeconomic status (SES). For SES definition, the guidelines of an Italian epidemiological study on a large population that involved a database from 18 Italian regions were used [24]. According to this study, SES measures included maternal and paternal education and employment [24].

All parents provided written informed consent to participate in this study. The investigation was carried out in accordance with the principles of the Declaration of Helsinki [25]. The Departmental Ethics Committee at the University of Campania approved the study.

#### 2.2.1. Polysomnographic (PSG) Data

PSG data were reviewed and analyzed visually from inpatient children between January and June 2016 at the Sleep Lab for Developmental Age of the University of Campania “Luigi Vanvitelli” in Italy to establish the presence of SRBD and inclusion in the OSAS group. The data were compared with PSG data obtained in the same Sleep Lab from other inpatients but were negative for SRBD collected between 2013 and 2015 and therefore considered normal and identified as non-OSAS (nOSAS).

Considering that all PSG data presented at least a desaturation level percentage (%) ≥ 3% and according to the current international guidelines for SRBD scoring and evaluation [26], the apnea/hypopnea (AHI/h) per hour was used as the measure of OSAS grading as follows: mild OSAS (AHI 1 to <5 events per hour), moderate OSAS (AHI ≥ 5 to <10 events/hour), and severe OSAS (AHI ≥ 10 events/hour). In the present study, we included only the severe OSAS group (AHI ≥ 10/hour).

#### 2.2.2. Bar-On Emotional Quotient Inventory: Youth Version (EQ-i:YV)

The Italian version of the *EQ-i:YV* was used [27]. It is a self-report measure for emotionally and socially intelligent behavior, providing and estimating the emotional and social intelligence. The self-report scale is composed of 60 items with a total emotional intelligence score and 4 subscales that generated a general emotional quotient (EQ) score: (1) Intrapersonal relationship that evaluates one's ability to understand and express his feelings and needs; (2) Interpersonal relationship that evaluates one's empathy towards others, his understanding of others' emotions, and the quality of his relation and interaction with others; (3) Stress Management that evaluates stress tolerance and impulse control; and (4) Adaptability that evaluates one's ability to adapt and regulate his emotions in different settings, and it is a combination of the EQ-i's flexibility reality testing and problem-solving scales, General Mood and Positive Impression. The validation of the inventory on North American samples suggests that the Bar-On Emotional Quotient Inventory: Youth Version has excellent psychometric properties and identifies core features of emotional intelligence in children. The survey presents an additional scale to help the interpretation of the results: a General Mood scale, a Positive Impression scale, and an Inconsistency Index. The test is widely used to explore a large range of psychological disorders among children and adolescents ranging from 8 to 18 years, and it presents good internal reliability (Cronbach's *α* for each subscale for females is between 0.82 and 0.90). The Bar-On EQ-i:YV uses a 4-point Likert-style format, with response options ranging from “very seldom true of me” (score 1) to “very often true of me” (score 4). Standard scores have a mean of 100, and a standard deviation of 15 scores below 90 is indicative of problematic levels of emotional intelligence, and the greater the number of scores below 90, the greater the likelihood that the results indicate a moderate to severe problem or deficiency. A standard score in the range between 90 and 110 indicates effective emotional and social functioning. A score greater than 110 suggests the presence of enhanced emotional and social skills, while a score of less than 90 suggests that emphasis should be placed on enriching skills in that area [27].

### 2.3. Statistical Analysis

A chi-square test and a *t*-test were performed when appropriate, in order to compare the two groups (OSAS and nOSAS) for age, gender, and EQ-i:YV scores. Moreover, Cohen's *d* analysis was performed in order to verify the effect size. According to Cohen, a score of 0.2 is indicative of a small effect, a score of 0.5 indicates a medium effect size, and a score of 0.8 or more indicates a large effect size. A *p* value ≤ 0.05 was considered statistically significant.

## 3. Results

Participants' demographic characteristics are shown in Table 1. The two groups were counterbalanced for gender [*χ*^2^_(1)_ = 0.437, *p* = 0.508], similar for age [*t* − test_(391)_ = 1.52, *p* = 0.129], height [*t* − test_(391)_ = 1.561, *p* = 0.119], weight [*t* − test_(391)_ = ‐1.125, *p* = 0.261], and BMI [*t* − test_(391)_ = 1.439, *p* = 0.151]. None of the participants had been treated pharmacologically or had any associated diseases.

Table 2 shows the comparisons for means and standard deviations between the severe OSAS group and the nOSAS group (Table 2) in nocturnal respiratory parameters. The OSAS group has shown significantly lower total sleep time (6.84 ± 1.25*vs.*8.24 ± 0.96) and oxygen mean saturation percentage (91.63 ± 1.39*vs.*97.15 ± 2.01) than the nOSAS group. Conversely, the OSAS group showed significantly higher means than the nOSAS group in the remaining nocturnal respiratory parameters, such as the apnea-hypopnea index/hour (13.84 ± 1.12*vs*. 0.64 ± 2.01), oxygen desaturation index/hour (10.19 ± 2.86*vs.*0.31 ± 0.21), and oxygen desaturation level percentage (7.89 ± 1.11*vs.*0.15 ± 0.12).

In addition, OSAS children showed lower scores compared to nOSAS children in several EQ-i:YV subscales such as Interpersonal (81.92 ± 6.34*vs*. 98.16 ± 7.81), Adaptability (79.61 ± 7.36*vs.*101.32 ± 4.09), Stress Management (75.12 ± 8.34 vs. 99.44 ± 3.12), and EQ Total (83.51 ± 10.78 vs. 103.12 ± 7.15), with statistically significant differences (*p* < 0.001) and a very large effect size (Table 3). In addition, the Intrapersonal (*p* = 0.456), General Mood (*p* = 0.733), and Positive Impression (*p* = 0.298) subscales did not show significant differences (Table 3).

**Figure 1:**
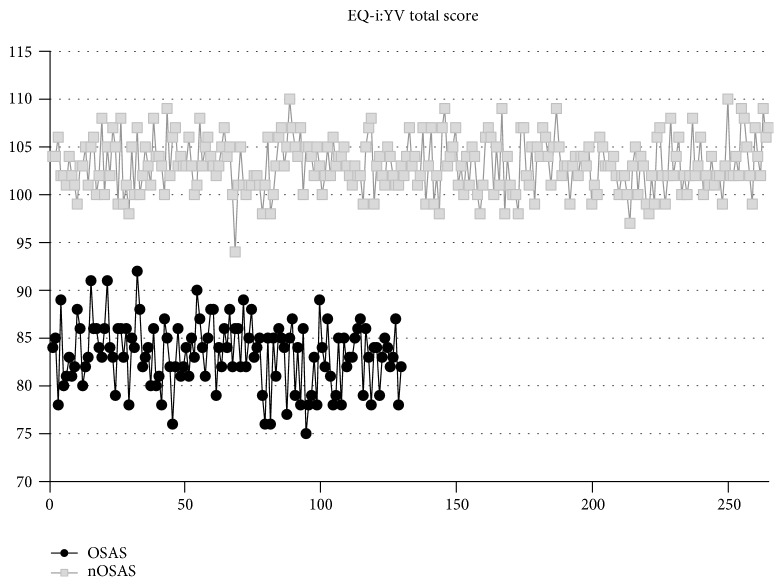
Raw data distribution of children affected by obstructive sleep apnea syndrome (OSAS) and non-OSAS (nOSAS) children in EQ-i:YV total score. EQ-i:YV: Bar-On Emotional Quotient Inventory: Youth Version.

Figure 1 shows the raw data on the EQ Total score in order to display the prevalence of subjects above and below threshold (the normal range was considered between 90 and 110 points, a score ≥ 110 points was considered above the threshold, and a score ≤ 90 points was considered below the threshold). Specifically, the OSAS group tends to have a distribution above 90 points.

## 4. Discussion

The present study investigated emotional intelligence in a large group of OSAS children, compared to typical developing peers. This study design started by a pilot study published in 2017 focused on the same topic with a smaller sample size recruited by Parisi et al. [28].

The Bar-On Emotional Quotient Inventory: Youth Version was used. OSAS children showed impaired results in the EQ Total and three out of four subscales: Interpersonal Relationship scale that assessed the quality of interpersonal relations and interactions and the ability to understand others' emotions, Adaptability scale that refers to the ability to adapt emotions in different settings, and Stress Management scale that evaluates stress tolerance and impulse control. OSAS children, therefore, compared to healthy peers, showed relevant difficulties in several emotional domains. Such problems may affect their social life and relationships.

According to these findings, then, it seems important that the physicians who deal with children with OSAS not only consider the specific problems of the respiratory pathology but also take into account the emotional implications that this event involves, especially since, according epidemiological data, respiratory disorders may be viewed as a public health problem affecting approximately 1% to 6% of all children, up to 59% of obese children, 2% to 24% of adults, and 70% of bariatric surgery patients. The incidence increases with age causing considerable direct and indirect health care and economic and social costs as well as human, in the form of motor vehicle crashes, medical conditions, including cardiovascular disease, metabolic syndrome, diabetes, and cerebrovascular disease, and perioperative morbidity and mortality [29].

In addition, OSAS has implications for job and academic performance and has been associated with potentially life-long cognitive impairment as well as sudden death. In this picture, cognitive alteration in memory domains has been showed in adults affected by severe OSAS particularly as greater susceptibility to false recognition [30], probably as direct effects of abnormalities in NREM sleep instability [31], or as specific proteomic biomarkers such as insulin and vascular signaling pathways such as prodromal for Alzheimer's disease [32] whereas ERP P300 and P300 means are considered to be electrophysiological measures that could be better indicators of cognitive changes than neuropsychological tests in OSAS, particularly in mildly affected patients [33–35].

OSAS has numerous comorbidities in children that can affect various aspects of life, not only for the subsequent disability but also especially for those still not well identified and remaining unmet such as the familiar stress, behavioral impairments, and cognitive alterations.

Moreover, OSAS contributes to cognitive deficits as supported by research showing impaired learning and behavioral problems in juvenile rats exposed to intermittent hypoxia during sleep, as well as by imaging studies showing cerebral neuronal injury in children with OSAS [36]. In this light, several areas of the PFC [e.g., the dorsolateral prefrontal cortex (DLPFC) and ventrolateral prefrontal cortex (VLPFC)] play an important role in the integration of emotion and cognition. Namely, MRI studies have revealed abnormalities in the DLPFC, VLPFC, and orbitofrontal cortex in patients with psychiatric conditions. In addition to the PFC, dispositional envy may also recruit the activation of regions related to the perception of emotions or intentions, such as the temporal gyrus [37].Some limitations of the study need to be acknowledged. The first limitation was the restricted age group studied. Further research including the measurement of a more extended age group population should investigate interactions between emotional intelligence skills and intermittent hypoxia due to the respiratory disorders. The second limitation was that the study did not have a follow-up that would allow a longitudinal assessment of the consequences of respiratory disorders. The third limit was the restricted time of the observational study and the quantity of variables detected. Further prospective studies with longer observational times, including the evaluation of other variables (i.e., duration of SRBD, cognitive level, executive functions, and emotional difficulties), would be needed to confirm and generalize our results.

In this perspective, our data may be interpreted highlighting the role of intermittent hypoxia in OSAS effect genesis, involving also aspects different from physical impairments, although further studies are needed, particularly in the developmental age in order to prevent the neuropsychological and neurocognitive effects described above.

## Figures and Tables

**Table 1:** Participants' demographic characteristics.

	OSAS (*n* = 129)	nOSAS (*n* = 264)	*t*/*χ*^2^	*p*
Age	7.64 ± 1.98	7.98 ± 2.13	0.437^#^	0.508
M/F	72/57	138/126	1.52^§^	0.129
Height	126.1 ± 1.68	126.4 ± 1.84	1.561^#^	0.119
Weight	27.8 ± 1.87	27.6 ± 1.54	-1.125^#^	0.261
*z*-BMI	0.38 ± 0.22	0.41 ± 0.18	1.439^#^	0.151

M: males; F: females; *z*-BMI: *z* scores of body mass index. Comparisons were made by independent sample *t*-test (^#^) and chi-square test (^§^).

**Table 2:** Comparisons for means and standard deviation (±) between the children affected by obstructive sleep apnea syndrome (OSAS) and non-OSAS (nOSAS) children in nocturnal respiratory parameters.

Nocturnal respiratory parameters	OSAS (*n* = 129)	nOSAS (*n* = 264)	*t*-test (df = 391)	*p*
Total sleep time (TST)	6.84 ± 1.25	8.24 ± 0.96	-12.252	<0.001^∗^
Apnea/hypopnea index (AHI)/hour	13.84 ± 1.12	0.64 ± 0.12	189.53	<0.001^∗^
Oxygen desaturation index (ODI)/hour	10.19 ± 2.86	0.31 ± 0.21	55.893	<0.001^∗^
Oxygen mean saturation percentage (%)	91.63 ± 1.39	97.15 ± 2.01	-28.075	<0.001^∗^
Oxygen desaturation level percentage (%)	7.89 ± 1.11	0.15 ± 0.12	112.111	<0.001^∗^

Nocturnal respiratory parameters: total sleep time (TST), apnea/hypopnea index (AHI)/hour, oxygen desaturation index (ODI)/hour, oxygen mean saturation percentage (%), and oxygen desaturation level percentage (%). ^∗^*p* values < 0.05 are statistically significant. df: degrees of freedom.

**Table 3:** Comparisons for means and standard deviation (±) between the children affected by obstructive sleep apnea syndrome (OSAS) and non-OSAS (nOSAS) children in EQ-i:YV scores.

EQ-i:YV	OSAS (*n* = 129)	nOSAS (*n* = 264)	*t*-test (df = 391)	*p*	Cohen's *d*
Interpersonal	81.92 ± 6.34	98.16 ± 7.81	-20.537	<0.001^∗^	2.283
Intrapersonal	90.51 ± 6.72	91.12 ± 8.01	-0.746	0.456	0.083
Adaptability	83.54 ± 8.91	101.32 ± 4.09	-27.122	<0.001^∗^	2.565
Stress Management	75.12 ± 8.34	99.44 ± 3.12	-41.812	<0.001^∗^	3.863
General Mood	90.01 ± 8.12	90.34 ± 9.44	-0.34	0.734	0.037
EQ Total	83.51 ± 10.78	103.12 ± 7.15	-21.45	<0.001^∗^	2.144
Positive Impression	88.59 ± 4.12	89.12 ± 5.01	-1.042	0.298	0.116
Inconsistency Index	<5	<5	—	—	—

EQ-i:YV: Bar-On Emotional Quotient Inventory: Youth Version; df: degrees of freedom. EQ-i:YV scores: Interpersonal, Intrapersonal, Adaptability, Stress Management, General Mood, Emotional Quotient (EQ) Total, Positive Impression, and Inconsistency Index. ^∗^*p* values < 0.05 are statistically significant.

## Data Availability

The data used to support the findings of this study are available from the corresponding author upon request.
